# Mediterranean Diet and Particulate Matter Exposure Are Associated With LINE-1 Methylation: Results From a Cross-Sectional Study in Women

**DOI:** 10.3389/fgene.2018.00514

**Published:** 2018-10-30

**Authors:** Martina Barchitta, Andrea Maugeri, Annalisa Quattrocchi, Germana Barone, Paolo Mazzoleni, Alfio Catalfo, Guido De Guidi, Maria Giovanna Iemmolo, Nunzio Crimi, Antonella Agodi

**Affiliations:** ^1^Department of Medical and Surgical Sciences and Advanced Technologies “GF Ingrassia”, University of Catania, Catania, Italy; ^2^Department of Biological, Geological and Environmental Sciences, University of Catania, Catania, Italy; ^3^Department of Chemical Science, Section of Photochemistry and Photobiology, University of Catania, Catania, Italy; ^4^Research Centre for the Analysis, the Monitoring and Methodology for Environmental Risk Assessment, University of Catania, Catania, Italy; ^5^Department of Clinical and Experimental Medicine, University of Catania, Catania, Italy

**Keywords:** epigenetics, dietary habits, air pollution, gene–diet interaction, cardiovascular disease, metabolic disorders, cancer, hypomethylation

## Abstract

Emerging evidence suggests that air pollution increases the risk of cardiovascular disease (CVD) and metabolic disorders, adding to the global burden of disease attributable to lifestyle and behavioral factors. Although long interspersed nucleotide elements 1 (LINE-1) methylation has been associated with these disorders, no studies have simultaneously examined the effects of diet and air pollution exposure on DNA methylation. Herein, we evaluated the association of particulate matter (PM with aerodynamic diameters of less than 10 mm) exposure and adherence to Mediterranean Diet (MD) with LINE-1 methylation. Healthy women (*n* = 299), aged 15 to 80 years, were enrolled in a cross-sectional study. Dietary data and adherence to MD were assessed by a Food Frequency Questionnaire (FFQ) and Mediterranean Diet Score (MDS). PM10 levels during 1-month before recruitment were recorded by monitoring stations and assigned to each woman based on their residential address and day of recruitment. LINE-1 methylation in blood samples was assessed by pyrosequencing and reported as percentage of 5-methylcytosine (5mC). The Mann–Whitney *U* test, Spearman’s rank correlation test and linear regression models were applied. Our results demonstrated, for the first time, an inverse association between adherence to MD and exposure to PM10 with LINE-1 methylation: while higher monthly PM10 exposure decreases LINE-1 methylation level (β = −0.121; *p* = 0.037), the adherence to MD increases it (β = 0.691; *p* < 0.001). MDS seemed to interact with PM10 levels (*p* = 0.002) on LINE-1 methylation, as such we confirmed that the effect of MD decreased with increasing PM10 levels (β = 0.657; *p* < 0.001 in the first tertile; β = 0.573; *p* < 0.001 in the second tertile; β = 0.551; *p* < 0.001 in the third tertile). Thus, we suggest that LINE-1 methylation is a possible mechanism underpinning environment-related health effects, and encourage further research to evaluate whether the adherence to the MD could counteract the negative effect of PM10 exposure.

## Introduction

Air pollution constitutes a global health hazard, which may activate several pathways involving oxidative stress, vascular dysfunction, and metabolic impairment. In fact, air pollution increases the risk of cardiovascular disease (CVD) and metabolic disorders, adding to the global burden of disease attributable to lifestyle and behavioral factors (Stansfeld, 2015). The effect of chronic environmental exposure is complex since it occurs within the framework of the cumulative impact of exposures over time (Münzel et al., 2017). This approach recognizes that environmental-related diseases are the result of the totality of a person’s environmental exposures, from all sources and routes, across the lifespan – the so-called exposome (Wild, 2012).

In the last decades, several lines of evidence suggested that molecular effects of environment might extend beyond the interaction with the DNA sequence (Reamon-Buettner et al., 2008; Baccarelli and Bollati, 2009). In this context, epigenetics - the study of heritable changes in gene expression that occur without changes in DNA sequence (Wolffe and Guschin, 2000) – provides opportunities to understand the mechanistic underpinnings of environment-related health effects (Angrish et al., 2018). Epigenetic signatures, including DNA methylation, histone modifications, histone variants, and chromatin remodeling, are dynamic in response to environmental signals, modifiable during cell differentiation and heritable in daughter cells (Hammoud et al., 2013). Among these, DNA methylation can be potentially modified by environmental and lifestyle factors, reprogramming the genome of exposed individuals and future generations (Burris and Baccarelli, 2014). DNA methylation can regulate gene transcription and chromosome stability via the addition of methyl groups to cytosine residues. In mammalian, 5-methylcytosines (5mC) represent 2–5% of all cytosines and are mainly found on CpG dinucleotides, also called CpG sites. Although hypermethylation of CpG sites, located in promoter regions, leads to decreased expression of the genes (Orphanides and Reinberg, 2002), more than 90% of CpG sites are located in transposable repetitive elements (Yang et al., 2004). Hypomethylation of transposable repetitive elements has been associated with chromosomal instability and aberrant genome function (Schulz, 2006; Slotkin and Martienssen, 2007). Methylation level of transposable sequences, including both long interspersed nucleotide elements 1 (LINE-1) and Alu sequences, has been used as a surrogate marker of global genomic DNA methylation (Yang et al., 2004). Although LINE-1 methylation is not an universally accepted marker of global methylation, aberrant methylation of these sequences was shown to be associated with cancer (Woo and Kim, 2012; Barchitta et al., 2014b), CVD (Baccarelli et al., 2010), and degenerative diseases (Maugeri et al., 2018a,b).

Exposure to air pollution, especially to particulate matter, has been linked to epigenetic changes by several epidemiological studies. For instance, a study by Tarantini et al. (2009) showed that long-term exposure to particulate matter with aerodynamic diameters of less than 10 mm (PM10) was negatively associated with Alu and LINE-1 methylation. Consistently, results from the Normative Aging Study demonstrated that exposure to black carbon, a marker of traffic particles, was also negatively associated with LINE-1 methylation (Baccarelli et al., 2009). These findings point out the need of further research to determine whether air pollution leads to LINE-1 methylation changes. However, since nutritional factors may modulate the response to environmental exposure, current environmental health research should incorporate nutrition and dietary practices. For instance, several classes of nutrients (i.e., folate, polyphenols, selenium, retinoid, fatty acids, isothiocyanates, and allyl compounds) can affect DNA methylation via different mechanisms (Ong et al., 2011). Among these, folate is a fundamental methyl donor for cellular replication and maintenance via modulating DNA methylation, synthesis, and repair. However, since current evidence is controversial (Moore et al., 2008; Choi et al., 2009; Zhang et al., 2011b, 2012; Ono et al., 2012; Piyathilake et al., 2012; Agodi et al., 2015a), its effect remains to be completely elucidated. Focusing on dietary patterns, the adherence to diet characterized by a high intake of vegetables and fruits has been associated with LINE-1 methylation (Zhang et al., 2011b; Agodi et al., 2015a; Delgado-Cruzata et al., 2015). Despite these findings, lack of evidence still exists about the effect of Mediterranean Diet (MD) – widely recognized as the optimal diet for disease prevention and global health – on LINE-1 methylation. To our knowledge, no studies have simultaneously examined the effects of diet and air pollution exposure in experimental models or humans. The questions that need to be addressed include the interactive effects of both factors on surrogate molecular markers of diseases. Given this scenario, we recently designed the present project, aiming to evaluate whether adherence to MD, as well as the intake of specific nutrients, might modulate the association between air pollution and methylation in healthy women living in Catania (Barchitta et al., 2017b). In previous studies, we demonstrated the effect of both diet and epigenetic mechanisms in several female physiological and pathological conditions (Agodi et al., 2014, 2015a,b; Barchitta et al., 2014a, 2017a,c, 2018). Herein, we evaluated the association of PM10 exposure and adherence to MD, 1 month before recruitment, with LINE-1 methylation, taking into account their potential interaction and the effect of socio-economic and lifestyle factors.

## Materials and Methods

### Study Design

Women referred to two clinical laboratories of Catania (Italy) were fully informed of the purpose and procedures and invited to participate in this cross-sectional study. This study was carried out in accordance with the recommendations of the involved institution. The protocol was approved by the ethics committee of the involved institution. All subjects gave written informed consent in accordance with the Declaration of Helsinki. The inclusion criteria were: (i) non-pregnant women (ii) with no current or previous self-reported history of severe diseases, as cancer, CVD, type 2 diabetes, neurodegenerative and autoimmune diseases (iii) who signed a written consent to participate in the study. Information on sociodemographic and lifestyle data were collected by trained epidemiologists using a structured questionnaire. Educational level was classified as low (primary school, i.e., ≤8 years of school) and high (high school education or greater, i.e., >8 years of school). Women were also classified as employed or unemployed (including students and housewives). Body mass index (BMI) was calculated as weight (kg) divided by height (m^2^) and classified based on criteria from the World Health Organization (1995). No exclusion criteria for BMI categories were applied.

### PM10 Exposure Assessment

In Catania, air pollutants are continuously measured by a network of five automatic monitoring sites located in the urban area. PM10 levels, recorded by the Ecology and Environment Office of the Municipality of Catania through these monitoring stations and available online as daily means, were assigned to each woman based on their residential address and day of recruitment. Briefly, monitoring stations and residential addresses were geocoded and PM10 measurements from the nearest monitoring station to residential address were assigned. For each woman, we recorded the daily mean of PM10 level the day of recruitment and back to 30 days before. We also calculated the monthly mean PM10 level referred to the month before recruitment. Missing values were imputed by an algorithm integrating the annual average of the incomplete series and the PM10 concentrations of the nearest and more correlated monitors (Cattani et al., 2010).

### Dietary Assessment

Dietary data were obtained by a 95-item semi-quantitative Food Frequency Questionnaire (FFQ), using the previous month as the reference period (Agodi et al., 2011). For each food item, women were asked to report the frequency of consumption and portion size. To estimate the amount of each food item and to minimize inaccuracies, an indicative photograph atlas was used. Frequencies of food consumption were classified into 12 categories, ranging from “almost never” to “two or more times a day.” The medium serving sizes were described by natural portions or standard weight and volume measures of the servings commonly consumed in the Italian population. Accordingly, portion size was classified into three categories: small (half a medium serving size), medium, and large (1.5 times or more than a medium serving size). The food intakes derived from the FFQ were calculated by multiplying the frequency of consumption with the daily portion size of each food group. Folate and total caloric intakes were calculated using the USDA Nutrient Database^1^ adapted to the Italian food consumption. Intake of folic acid from supplements was specifically addressed as previously described (Agodi et al., 2014). Prevalence of folate deficiency was estimated by comparing folate intake with the Estimated Average Requirements (EAR) (Institute of Medicine (US) Food and Nutrition Board, 1998), taking into account the use of folic acid supplements.

### Mediterranean Diet Score

Adherence to MD was assessed using the Mediterranean Diet Score (MDS) (Trichopoulou et al., 1995; Couto et al., 2011) which includes 9 components: fruits and nuts, vegetables, legumes, cereals, lipids, fish, dairy products, meat products, alcohol and the ratio of unsaturated to saturated lipids. For components that are more consumed in Mediterranean countries (vegetables, legumes, fruits and nuts, cereals, fish, and a high ratio of unsaturated to saturated lipids), women whose consumption was below or equal to the median value of the population were assigned a value of 0, and a value of 1 was assigned otherwise. For components consumed less frequently in Mediterranean countries (dairy and meat products), women whose consumption was below the median were assigned a value of 1, and a value of 0 was assigned otherwise. A value of 1 was given to women consuming a moderate amount of alcohol (5 to <25 g per day). Accordingly, MDS ranges from 0 (no-adherence) to 9 (perfect adherence). MD adherence was categorized, according to the MDS, as follows: low adherence (MDS range: 0–3), medium adherence (MDS range: 4–6), or high adherence (MDS range: 7–9) (Barchitta et al., 2014a).

### DNA Extraction and Methylation Analysis

Whole blood samples, collected into EDTA tubes from each participant, were centrifuged at 2500 rpm for 15 min. The buffy coat fraction was transferred to a cryovial and immediately frozen at −20°C until use. DNA was extracted using the QIAamp DNA Mini Kit (Qiagen, Italy) according to the manufacturer’s protocol. LINE-1 methylation levels were measured by pyrosequencing-based methylation analysis, using the PyroMark Q24 instrument (Qiagen, Italy), as previously reported (Agodi et al., 2015a; Barchitta et al., 2017c). Briefly, bisulfite conversion and clean-up of DNA for methylation analysis of 30–40 ng of DNA were completed using the EpiTect Bisulfite Kit (Qiagen, Italy) and the converted DNA was eluted in 20 μl of Elution Buffer. PCR was conducted in a reaction volume of 25 μl, using the PyroMark PCR Kit (Qiagen, Italy). According to the manufacturer’s instructions, each reaction mixture contained 1.5 μl of bisulfite-converted DNA, 12.5 μl of PyroMark PCR Master Mix 2×, 2.5 μl of Coral Load Concentrate 10×, and 2 μl of the forward primer (5′-TTTTGAGTTAGGTGTGGGATATA-3′) and the reverse-biotinylated primer (5′-biotin-AAAATCAAAAAATTCCCTTTC-3′) (0.2 μM for each) (Agodi et al., 2015b). Hot start PCR cycling conditions were 1 cycle at 95°C for 15 min, 40 cycles at 94°C for 30 s, 50°C for 30 s, and 72°C for 30 s, and a final extension at 72°C for 10 min. Then, the PCR product underwent pyrosequencing using 0.3 mM of the sequencing primer (5′-AGTTAGGTGTGGGATATAGT-3′). All runs included 0 and 100% methylated human DNA as positive controls as well as a negative control. To confirm reproducibility every sample was tested two times and failed assays were repeated. Overall, intra-observer coefficient of variability between the two replicates of LINE-1 methylation measurements was 3.2% (SD = 3.0%). LINE-1 methylation levels were calculated as percentage of methylated cytosines over the sum of methylated and unmethylated cytosines, and reported for each CpG site as well as the average of the three CpG sites (GenBank Accession No. X58075).

### Statistical Analyses

Statistical analyses were performed using the SPSS software (version 22.0, SPSS, Chicago, IL, United States). Descriptive statistics were presented as frequencies, means ± standard deviations (SDs), median values and interquartile range (IQR). Prior to analysis, the normal distribution of all variables was checked using the Kolmogorov–Smirnov test. Since LINE-1 methylation exhibited non-normal distribution, differences by population characteristics were tested using the Mann–Whitney *U* test or the Kruskal–Wallis test. Correlation of daily and monthly PM10 levels with LINE-1 methylation was evaluated using the Spearman’s rank correlation coefficient and presented as a correlation matrix. To evaluate the association of monthly PM10 levels or MDS with LINE-1 methylation, we used the following linear regression models: the age-adjusted model; the multivariable model adjusted for covariates that were selected *a priori* (i.e., age, educational level, employment status, smoking, BMI, season of recruitment, dietary folate intake and use of supplements). In linear regression models, continuous variables, i.e., LINE-1 methylation level, PM10 level, MDS, age, BMI and dietary folate intake were used. Interaction of PM10 levels and MDS with LINE-1 methylation was investigated by using the general linear model, adjusting for age, educational level, employment status, smoking, BMI, and folate deficiency. Since interaction was statistically significant, we tested the association of MDS with LINE-1 methylation by using a multivariable-adjusted model stratified by tertiles of PM10 exposure. Correction for multiple comparisons was performed by the Bonferroni method and adjusted *p*-values were calculated by multiplying the unadjusted *p*-value by the number of comparisons. Overall, results were reported as beta regression coefficient (β) expressing the change in LINE-1 methylation level associated with a standard deviation increase in PM10 levels or MDS. A *p*-value of 0.05 was considered as statistically significant.

## Results

The main characteristics of the 299 women, aged from 15 to 80 years, included in the present study are given in Table 1. In summary, mean age was 38.99 years (*SD* = 16.53; median = 35), 69.9% reported high educational level and 54.2% were unemployed. With regard to lifestyle factors and nutritional status, 21.2% were current smokers and 37.9% were overweight or obese. Mean folate intake was 295.36 μg/day, and only 18.1% reported the use of folic acid supplements. Taking into account the use of supplements, prevalence of folate deficiency was 47.5%.

**Table 1:** Characteristics of study population.

Population characteristics (*N* = 299)	Mean *(SD)* or proportion
Age, years	38.99 (16.53)
Low educational level	30.1%
Unemployed	54.2%
Current smokers	21.2%
BMI, kg/m^2^	24.55 (5.04)
Underweight	4.7%
Normal Weight	57.4%
Overweight	24.0%
Obese	13.9%
Dietary folate intake, μg/day	295.36 (132.62)
Folic acid supplement users	18.1%
Folate deficiency	47.5%
MDS	4.64 (1.78)
Low adherence	27.1%
Medium adherence	58.2%
High adherence	14.7%
LINE-1 methylation level, % 5mC	67.66 (7.48)
CpG site 1, % 5mC	80.47 (3.14)
CpG site 2, % 5mC	57.11 (9.20)
CpG site 3, % 5mC	65.39 (7.48)

*SD, standard deviation; BMI, body mass index; 5mC, 5-methylcytosine.*

**FIGURE 1:**
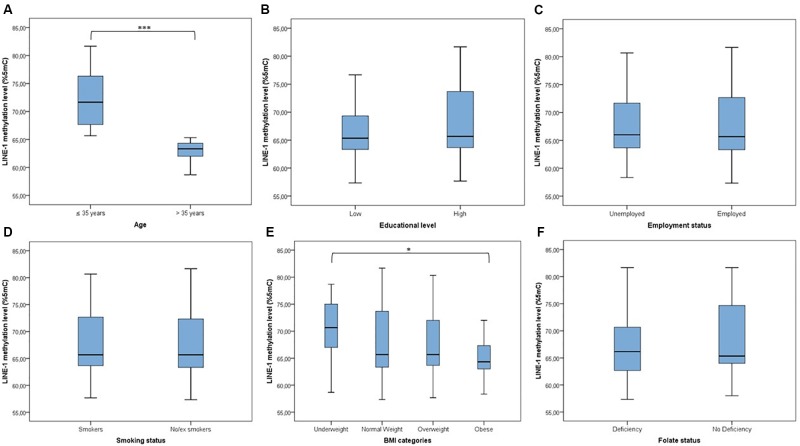
Distribution of LINE-1 methylation by population characteristics. **(A)** ≤Median age (35 years) vs. >median age; **(B)** low educational level (≤8 years of school) vs. high educational level (>8 years of school); **(C)** unemployed vs. employed (including part-time and full-time); **(D)** smokers vs. no/ex-smokers; **(E)** comparison across BMI categories; **(F)** folate deficiency vs. no folate deficiency (taking into account the use of supplements). ^∗∗∗^*p* < 0.001 and ^∗^*p* < 0.05 based on the Mann–Whitney *U* test or the Kruskal–Wallis test.

LINE-1 methylation levels at the three CpG sites correlated with each other (*r*_1–2_ = 0.398; *r*_1–3_ = 0.940; *r*_2–3_ = 0.394; *p* < 0.001), showing mean values of 80.47 (*SD* = 3.14), 57.11 (*SD* = 9.20) and 65.39 (*SD* = 7.48), respectively. The mean LINE-1 methylation level was 67.66 (*SD* = 7.48). Figure 1 shows that distribution of LINE-1 methylation levels differed by age (*p* < 0.001) and BMI categories (*p* = 0.047), while no significant differences by educational level, employment status, smoking and folate deficiency were evident.

**FIGURE 2:**
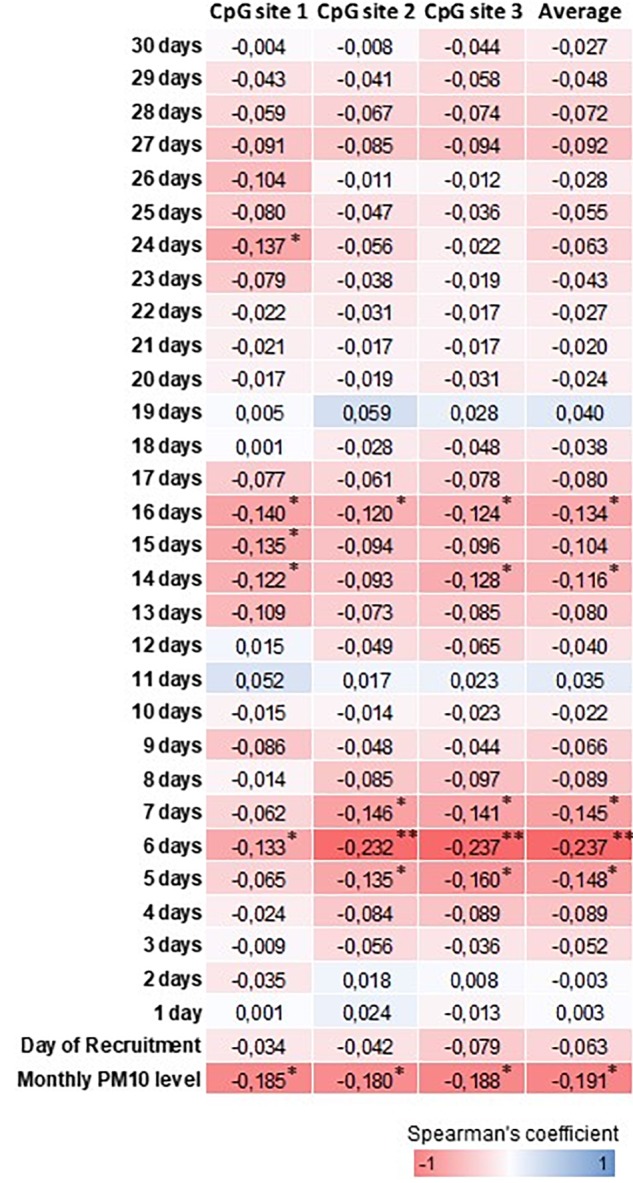
Heatmap representation of Spearman’s correlation coefficients between PM10 and LINE-1 methylation levels. ^∗∗∗^*p* < 0.001, ^∗∗^*p* < 0.01 and ^∗^*p* < 0.05.

**FIGURE 3:**
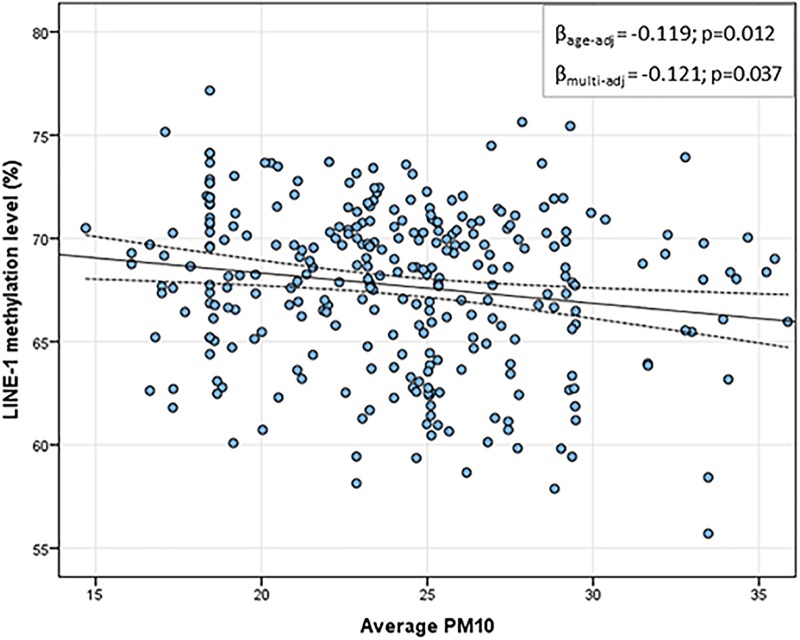
Scatter plot of the linear association between monthly mean PM10 and LINE-1 methylation levels. In the *x*-axis the monthly average PM10 levels; in the *y*-axis the predicted LINE-1 methylation level adjusted for age, educational level, employment status, smoking, BMI, season of recruitment, dietary folate deficiency and use of supplements.

Table 2 shows the daily PM10 levels attributed to each woman and evaluated during the 30 days before recruitment. PM10 levels ranged from 22.28 (8 days before recruitment) to 27.50 ng/m^3^ (20 days before recruitment). Accordingly, the monthly mean PM10 level was 24.18 ng/m^3^ (*SD* = 4.29). In order to assess whether PM10 levels were associated with LINE-1 methylation levels, we first analyzed the correlation between daily PM10 levels, as well as the monthly mean, and LINE-1 methylation levels. Figure 2 shows that PM10 levels during 16, 14, 7, 6, and 5 days before recruitment were weakly but significantly correlated with LINE-1 methylation levels. Consistently, a negative significant correlation with monthly mean PM10 level was shown. Scatter plot representing the linear association of monthly mean PM10 level with LINE-1 methylation is shown in Figure 3. In the age-adjusted model, monthly mean PM10 level was significantly and negatively associated with LINE-1 methylation (β = −0.119; *p* = 0.012). A negative association was also observed in the multivariable regression analysis (β = −0.121; *p* = 0.037), adjusting for age, educational level, employment status, smoking, BMI, season of recruitment, dietary folate deficiency and use of supplements. Interestingly, this model also showed that LINE-1 methylation was negatively associated with age (β = −0.519; *p* < 0.001) and dietary folate intake (β = −0.111; *p* = 0.001).

**Table 2:** Daily mean PM10 levels during 30 days before recruitment.

Days before recruitment	Mean	*SD*	Median	IQR (percentiles)	
				25th	75th
30 days	24.26	9.06	23.00	18.44	29.20
29 days	24.38	10.15	23.00	18.44	29.60
28 days	25.16	10.00	23.70	19.11	29.20
27 days	24.54	9.16	23.50	18.44	29.10
26 days	25.17	11.61	23.00	18.60	28.60
25 days	25.04	11.02	23.00	18.44	28.60
24 days	24.07	9.63	21.29	18.44	28.60
23 days	24.32	8.84	23.60	18.44	29.10
22 days	25.32	11.53	23.80	18.44	29.20
21 days	26.52	12.60	24.20	18.44	31.90
20 days	27.50	15.84	23.90	18.44	31.30
19 days	27.17	15.80	23.00	18.44	30.70
18 days	25.44	11.33	22.70	18.44	28.60
17 days	24.90	9.66	23.00	18.44	29.10
16 days	24.39	9.07	22.00	18.44	30.20
15 days	23.73	8.42	22.00	18.44	27.70
14 days	23.83	8.40	21.60	18.44	29.10
13 days	24.03	8.45	21.60	18.44	27.80
12 days	23.97	8.13	21.80	18.44	27.80
11 days	23.70	8.80	20.70	18.44	27.60
10 days	23.67	8.72	21.00	18.44	29.20
9 days	23.16	8.32	21.80	18.44	27.70
8 days	22.28	7.52	21.30	18.44	25.20
7 days	22.64	7.29	20.70	18.44	26.50
6 days	22.75	7.48	20.47	18.44	27.70
5 days	23.00	10.11	20.47	18.44	26.60
4 days	22.39	7.66	20.47	18.44	25.70
3 days	23.51	7.43	21.30	18.44	27.60
2 days	22.77	7.91	20.70	18.44	26.20
1 day	22.66	8.45	20.50	18.44	26.10
Day of recruitment	23.39	8.59	21.10	18.44	27.30

*SD, standard deviation; IQR, interquartile range.*

**FIGURE 4:**
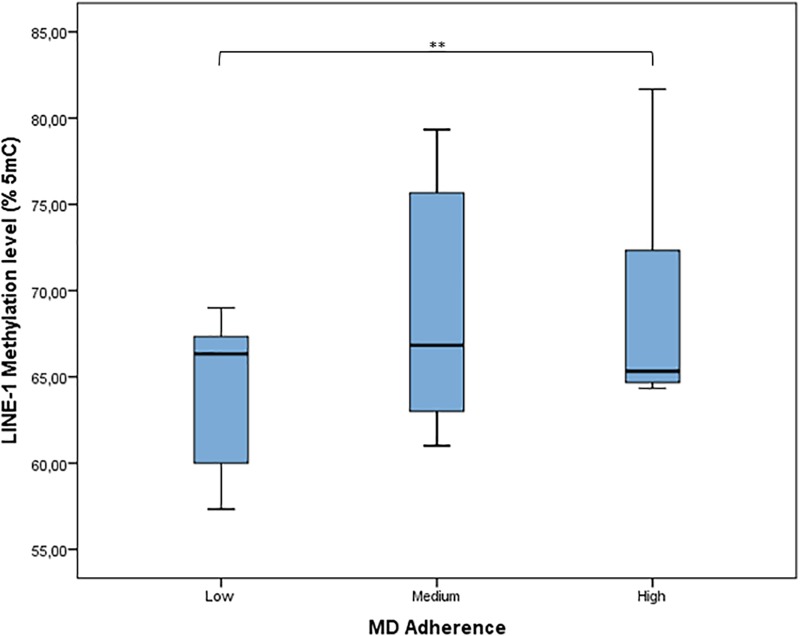
Distribution of LINE-1 methylation by categories of adherence to MD. MD adherence was categorized, according to the MDS, as low adherence (MDS range: 0–3), medium adherence (MDS range: 4–6), or high adherence (MDS range: 7–9). ^∗∗^*p* < 0.01 based on the Kruskal–Wallis test.

According to MDS (mean = 4.64; *SD* = 1.78), adherence to MD has been classified as low (27.1% of women), medium (58.2% of women), and high (14.7% of women). The distribution of LINE-1 methylation by categories of adherence to MD shows that LINE-1 methylation levels increased with increasing MD adherence (*p* = 0.004) (Figure 4). Results from linear regression analysis demonstrated that MDS was significantly and positively associated with LINE-1 methylation levels both in the age-adjusted (β = 0.606; *p* < 0.001) and multivariable-adjusted model (β = 0.691; *p* < 0.001). By contrast, age was significantly and negatively associated with LINE-1 methylation in both models (β = −0.787; *p* < 0.001; β = −0.581; *p* < 0.001).

In order to assess whether the adherence to MD could influence the relationship between PM10 exposure and LINE-1 methylation, we tested the interaction between MDS and monthly mean PM10 level. Since MDS seemed to interact with PM10 levels (*p* = 0.002), we conducted a multivariable linear regression analysis stratified by tertiles of PM10 exposure. Our results confirmed that MDS was significantly and positively associated with LINE-1 methylation, and that its effect decreased with increasing PM10 levels (β = 0.657; *p* < 0.001 in the first tertile; β = 0.573; *p* < 0.001 in the second tertile; β = 0.551; *p* < 0.001 in the third tertile).

## Discussion

Several lines of evidence indicate that dynamic changes in LINE-1 methylation – a well-established event in cancer and CVD (Baccarelli et al., 2010; Woo and Kim, 2012; Barchitta et al., 2014b) – appear to be influenced by environmental and lifestyle risk factors. In this framework, our findings support the relationship between air pollution exposure and epigenetic changes (Baccarelli and Bollati, 2009; Burris and Baccarelli, 2014). In line with previous work (Baccarelli et al., 2009; Tarantini et al., 2009; Madrigano et al., 2011), we observed that exposure to PM10 is negatively associated with LINE-1 methylation in healthy women. The study by Tarantini et al. (2009) conducted in an electric furnace steel plant, demonstrated that PM10 levels impacted on genomic DNA methylation of workers over an extended time frame. In particular, they reported that long-term, but not short-term, exposure to PM10 was inversely associated with methylation of Alu and LINE-1 sequences (Tarantini et al., 2009). This evidence, though from a different setting with peculiar exposure, led us to evaluate the correlation of 1-month daily PM10 exposure with LINE-1 methylation. Particularly, we observed that the effect was maximum for the exposure of 6 days before recruitment and spanned from days 5 to 7. Since a negative correlation with monthly mean PM10 level was also evident, we tested the association of monthly PM10 exposure and LINE-1 methylation. Notably, we demonstrated, for the first time, that monthly mean PM10 level was significantly and inversely associated with LINE-1 methylation both in the age- and multivariable-adjusted model.

In our analysis, we also observed that age was negatively associated with LINE-1 methylation. Although some studies reported no effect of aging on LINE-1 methylation (Chalitchagorn et al., 2004; El-Maarri et al., 2011; Zhang et al., 2011a), our result was in line with previous works, which demonstrated that methylation levels in the repetitive elements, including LINE-1, significantly decreased with increasing age (Bollati et al., 2009; Zhu et al., 2012; Cho et al., 2015). However, the observed effect of age on LINE-1 methylation appeared stronger if compared to evidence from previous similar investigations.

Several scientific evidences also suggested that dietary folate status, as well as amounts of other methyl donors, could affect global DNA methylation. However, these findings were conflicting: while the majority of studies demonstrated that global DNA methylation levels increased with increasing folate intake (Moore et al., 2008; Choi et al., 2009; Zhang et al., 2011b, 2012; Piyathilake et al., 2012; Agodi et al., 2015a), others observed an inverse relationship (Ono et al., 2012). This controversy might be explained by differences in unmeasured factors such as ethnicity, genetic variants in one-carbon metabolism, lifestyles, physiological and pathological conditions, which in turn can affect DNA methylation process.

The study of dietary patterns plays an important role in evaluating causes and consequences of PM exposure for human health and disease. This concept raised from the need of a more complete environmental exposure assessment in epidemiological studies, providing a comprehensive description of lifelong exposure history (Wild, 2012). In this context, there is growing interest in determining how dietary patterns may affect global and gene-specific DNA methylation. To our knowledge, the present study was the first to demonstrate that adherence to MD was positively associated with LINE-1 methylation levels, after adjusting for age, educational level, employment status, smoking, BMI, season of recruitment, dietary folate deficiency and use of supplements. This was consistent with previous studies showing that healthy women with high intake of vegetables and/or fruits had a lower risk of LINE-1 hypomethylation (Zhang et al., 2011b; Agodi et al., 2015a). The biological basis of this relationship could be attributed to the wide variety of nutrients and bioactive compounds provided by MD, such as phytochemicals (phenolics, flavonoids, and carotenoids), vitamins (vitamin C, folate, and pro-vitamin A), minerals (potassium, calcium, and magnesium), and fibers, which in turn act on multiple signal transduction pathways and epigenetic mechanisms (Saura-Calixto and Goñi, 2009; Liu, 2013).

Since we demonstrated the inverse association of exposure to PM10 and adherence to MD with LINE-1 methylation levels, we finally aimed to assess whether adherence to MD could influence the relationship between monthly PM10 levels and LINE-1 methylation. Our findings confirmed not only the interaction between MDS and PM10 exposure, but also that the positive effect of MD on LINE-1 methylation decreased with increasing PM10 levels.

This study had some limitations. Although hypomethylation of transposable repetitive elements, including LINE-1 sequences, has been associated with chromosomal instability and aberrant genome function (Schulz, 2006; Slotkin and Martienssen, 2007) and with different chronic degenerative diseases (Baccarelli et al., 2010; Woo and Kim, 2012; Barchitta et al., 2014b; Maugeri et al., 2018b), mechanisms of DNA hypomethylation are not fully understood. Therefore, the cross-sectional design of our analysis arises the need of further prospective studies and of functional test to explore the causes and the consequences of these modifications and to better understood the biological significance of LINE-1 methylation alterations. Moreover, the peculiar geological and environmental conditions, due to natural sources of particles from the near Mount Etna volcano, sea salt and Sahara dust could be a potential confounder in our study. Although PM10 exposure assessment method has been used in other studies (Cattani et al., 2010; De Prins et al., 2013), this method has not been previously validated in our population characterized by specific PM exposure. Thus, to validate the PM10 exposure assessment method, including both volcanic and usual urban PM exposure, further research should develop a standardized protocol to characterize the mineralogical-chemical composition of PM and to better define exposure. In addition, limiting our investigation PM10 exposure avoided potential concerns related to the effect of other environmental and work-related chemicals that modify epigenetic marks, including metals (cadmium, arsenic, nickel, chromium, and methylmercury), air pollutants (PM2.5, black carbon, and benzene), and toxicants (diethylstilbestrol, bisphenol A, persistent organic pollutants, and dioxin). Other potential weaknesses regard the dietary assessment and the observed relationship between MD and LINE-1 methylation. Although dietary assessment through FFQ does not preclude measurement errors and inaccuracies, the FFQ used in the present study has been previously developed and validated for use among our population (Agodi et al., 2011). However, the relationship between MD and LINE-1 methylation may be also affected by genetic factors (i.e., polymorphisms in the *MTHFR* gene), which in turn may interact with folate status and methylation process (Agodi et al., 2015c). With regard to molecular analysis, precision and reproducibility of the DNA methylation assay should be considered when interpreting results of the present study. Reliability and flexibility have made pyrosequencing of bisulfite-treated DNA the “gold standard,” and a high-throughput and replicable methodology to evaluate LINE-1 methylation (Beck and Rakyan, 2008; Rakyan et al., 2011). However, in the present study, the methylation analysis was performed on white blood cell DNA, including several cell type subsets. Previous studies reported small differences in LINE-1 methylation levels according to target CpG site and blood cell composition (Nelson et al., 2011; Zhu et al., 2012; Koestler et al., 2013; Tarantini et al., 2013; Nüsgen et al., 2015). Thus, the distinctiveness of LINE-1 methylation patterns discourages the comparison between results from studies, which evaluated LINE-1 methylation status at different CpG sites and reinforces the importance of accounting for cellular heterogeneity in future research.

## Conclusion

In conclusion, results of the present study, conducted in a sample of healthy women, demonstrate the inverse association of adherence to MD and exposure to PM10 with LINE-1 methylation levels. Moreover, our findings suggest that LINE-1 methylation is a possible mechanism underpinning environment-related health effects and thus, further research are encouraged to evaluate whether the adherence to the MD could counteract the negative effect of PM10 exposure.

## Author Contributions

AA is the principal investigator of the project. AA, GB, PM, GDG, and NC conceived and designed the research. MB, AM, and AQ performed the experiments and statistical analyses. MB, AM, and AA wrote the original draft. All the authors reviewed, edited, and approved the final draft.

## Conflict of Interest Statement

The authors declare that the research was conducted in the absence of any commercial or financial relationships that could be construed as a potential conflict of interest. The handling Editor declared a past co-authorship with one of the authors AA.

## Footnotes

1 http://ndb.nal.usda.gov/
